# Combined Rifampin and Sulbactam Therapy for Multidrug-Resistant Acinetobacter Baumannii Ventilator-Associated Pneumonia in Pediatric Patients

**DOI:** 10.24015/JAPM.2018.0072

**Published:** 2018-07

**Authors:** Jinlan Chen, Yifeng Yang, Kun Xiang, David Li, Hong Liu

**Affiliations:** 1 Department of Cardiovascular Surgery, The Second Xiangya Hospital, Central South University, Changsha, China;; 2 Department of Anesthesiology and Pain Medicine, University of California Davis Health, Sacramento, CA, USA.

## Abstract

**Background:**

With essentially no drug available to control the infection caused by the extensively drug-resistant Acinetobacter baumannii (XDR-Ab) in infants and young children, this study explored the clinical outcomes of pediatric patients with drug-resistant XDR-Ab who were treated with rifampicin in combination with sulbactam sodium.

**Methods:**

The data for clinical outcomes, microbiological responses, and side effects were collected and evaluated for 12 critically ill infants and young children diagnosed with ventilator-associated pneumonia caused by XDR-Ab following surgical treatment for congenital heart disease in a pediatric cardiac intensive care unit. This study was approved by local institutional review board (IRB).

**Results:**

Two patients died from the complex underlining diseases. The other 10 patients were weaned off the mechanical ventilation successfully within 4–15 days after the start of treatment with rifampicin combined with sulbactam sodium and discharged home. Three cases experienced adverse side effects, including severe rash and elevated aminotransferase level.

**Conclusion:**

The combination of rifampicin and sulbactam sodium appeared to be an effective and safe therapy for severe ventilator-associated pneumonia caused by XDR-Ab in infants and young children. Side effects such as skin rashes and elevated aminotransferase levels can be reversed once rifampicin is discontinued in time. (Funded by the Department of Cardiovascular Surgery, The Second Xiangya Hospital, Central South University, Chang­sha, China; the Departments of Anesthesiology and Pain Medicine of University of California Davis Health; and the National Institutes of Health.)

Acinetobacter baumannii (Ab) is one of the most common gram-negative bacteria in nosocomial infection. Extensively drug-resistant Ab (XDR-Ab) has become a global challenge because of its resistance to treatment and high mortality. Therefore, efforts have been made to find the cure for the infection of this XDR-Ab in the past decade (1–4). The combinations of rifampicin with colistin, carbapenem, or sulbactam sodium were preferred. When encountered with an XDR-Ab infection, a difficult choice of treatment is faced. In vitro experiments, animal studies, and clinical reports have shown that combined use of drugs can effectively control the XDR-Ab infection (2, 5–7). However, the clinical experiences were primarily limited to adult patient population, the clinical application of rifampicin combined with sulbactam sodium in infants and children has been rarely reported. Here, we present our experience using rifampicin combined with sulbactam sodium in the treatment of ventilator-associated pneumonia (VAP) caused by severe XDR-Ab infection after surgery for congenital heart disease (CHD) in infants and young children.

## METHODS

**Figure 1.**
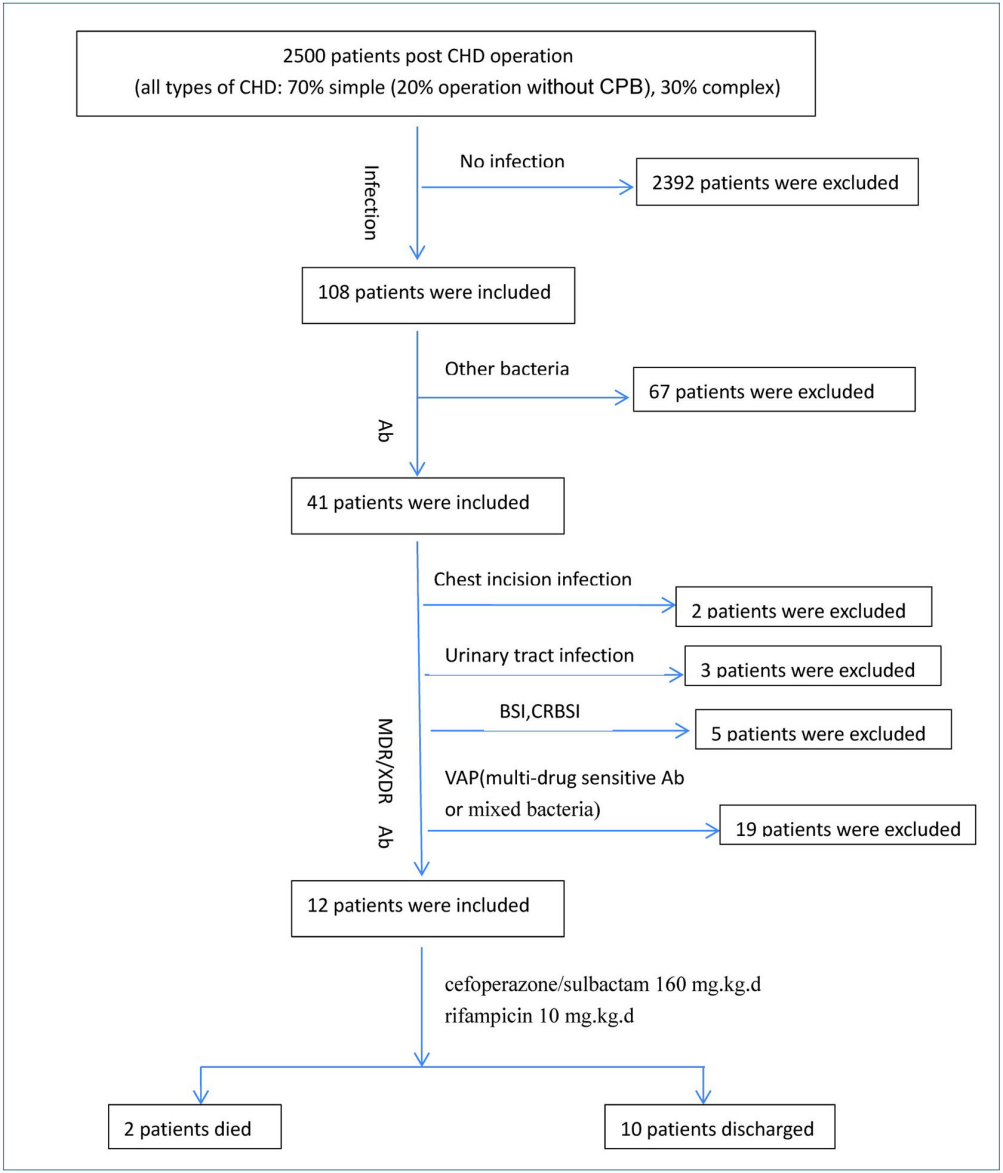
The Flow Chart of Patient Selections. Ab, Acinetobacter baumannii; MDR, multidrug-resistant; XDR, extensively drug resistant; VAP, ventilator-associated pneumonia; BSI, bloodstream infection; CRBSI, catheter related bloodstream infection.

Medical records of more than 2, 500 patients who were hospitalized in the pediatric cardiac intensive care unit (PCICU) following surgical treatment for CHD from June 2014 to June 2016 were retrospectively analyzed. Of these, 108 patients were diagnosed with infections, of which 41 patients were colonized/infected with Ab. The patients with mixed infections, infections in other tissues, or infections sensitive to antibiotics were excluded. The final study population included 12 critically ill pediatric patients with VAP caused by multi-drug resistant(MDR)/ XDR / pan-drug resistant (PDR) Acinetobacter baumannii (MDR/XDR/PDR-Ab) infection (3 pediatric patients simultaneously had bloodstream infection) (Figure 1 and Table 1). The diagnosis of clinical infection was based on clinical symptoms and bacteriological culture. All 12 patients met the diagnostic criteria for VAP: all patients had diagnostic conditions, such as long ventilator support time, fever, high white blood cell count, and more viscous secretions in the respiratory tract, and imaging examination in all patients showed infiltrating shadows within the lungs. Moreover, MDR-Ab was detected in cultures of tracheal secretions or bronchoalveolar lavage fluid. This study was approved by the medical ethics committee of the Second Xiangya Hospital of Central South University, Hunan, China.

In the patient whose ventilator could not be withdrawn within a short-term after CHD surgery, the secretion in the airway was sucked or the alveolar lavage fluid was collected using a bronchofiberscope for culture every day for 3 consecutive days from post-operation day 2. Peripheral blood culture was carried out three times for patients with high white blood cell counts (> 11, 000 / mcl) and high fever (> 38.5oC). If there was redness or purulent exu­date at the puncture site of the deep vein catheter, the deep vein catheter was removed and the front end was sent for culture along with simultaneous peripheral blood culture. All specimens were sent to the hospital laboratory where they were tested with reference to NCCLS antimicrobial susceptibility testing standards. A WalkAway 96 automatic bacterial identification and antimicrobial susceptibility testing system (Dade Beh­ring, USA) was employed. Quality-control strains were supplied by the National Center for Clinical Laboratories. The antimicrobial agents used to test the sensitivity to Ab were: ampicillin/ sulbactam, ticarcillin / clavulanic acid, amikacin, aztreonam, ceftazidime, cefotaxime, ciprofloxacin, cefepime, cefoperazone/sulbactam, gentamicin, imipenem, levofloxacin, meropenem, minocycline, piperacillin/tazobactam, co-trimoxazole, and tigecycline (Table 2).

Patients without preoperative pulmonary infection were given class I or II cephalosporin antibiotics postoperatively to prevent infection as standard, and close observation of body temperature along with blood examination and chest radiography were carried out routinely. The patients with symptoms of infection were empirically treated with cefoperazone/sulbactam sodium (100mg/kg/day intravenously every 8 hours) If the results of secretion or blood culture indicated XDR-Ab infection with resistance to carbapenems, the patients were treated with cefoperazone / sulbactam combined with rifampicin according to the results of drug sensitivity test. We used sulperazone (cefoperazone/sulbactam 1: 1, Pfizer) with a sufficient dosage; the dosage for pediatric patients infected with drug-resistant bacteria or bacteria that tended to be drug-resistant was 160 mg/kg/day, which was administered in four fractions every 6 hours through intravenous infusion. Meanwhile, rifampicin at a dose of 10 mg/kg/day was administered in two fractions every 12 hours through intravenous infusion. Attention was paid to the skin over the whole body and liver function during the drug application, and rifampicin was immediately stopped in patients with serious adverse drug reactions. Otherwise, rifampicin was used until the infection indexes were controlled, the white blood cell count normalized, the sputum volume was reduced, the imaging results were improved, and the patient was successfully weaned off the ventilator or even microbiological clearance was achieved.

In addition to antibiotic therapy, the following treatments were applied: positive inotropic drugs were used to support cardiac function; physical therapy was begun in all children after the hemodynamics were stable and the expecto­ration drainage was promoted; immunoglobulin at 300 mg/kg/day was routinely used for 3 days; enteral nutrition was provided while the gastrointestinal condition and daily calorie amounts were assessed and the nutrition volume was gradually increased; and the liquid intake and output were monitored hourly with a negative liquid balance goal in the early stage after surgery and a positive liquid balance allowed to some extent in the middle and advanced stages after surgery to ensure tissue repair.

## RESULTS

All 12 critical pediatric patients after surgical treatment for CHD had MDR/XDR/PDR Ab infection due to extended ventilator support time, an extended stay in the ICU, and complicated underlying conditions. The mean age of the 12 patients was 466 days, and the mean weight was 8.5 kg (Table 1). In case 1, the pathogenic bacteria in the culture of the internal jugular vein catheter were consistent with those in the blood culture, and the patient was diagnosed with catheter-related bloodstream infection (CRBSI). Two other blood cultures showed positive results in cases 7 and 8, and both patients were diagnosed with bloodstream infection (BSI). Three patients had multiple extracardiac malformations (Noon-an’s syndrome in case 4, the bilateral diaphragmatic defect with post abdominal wall defect repair in case 7, and Down’s syndrome in case 11).

Three patients had preoperative heart failure and pneumonia that was difficult to control. The neonate in case 2 underwent emergency surgery due to an obstructed total anomalous pulmonary venous connection (TAPVC). Three patients had severe airway stenosis, and four patients had low cardiac output after surgery. Case3 underwent peritoneal dialysis due to renal failure, and case 9 underwent extracorporeal membrane oxygenation (ECMO) to assist circulation due to severely low cardiac output after tricuspid valve replacement (TVR). Case 5 had severe postoperative laryngeal edema and stayed in the ICU for 2 weeks. Case 12 underwent repeated endotracheal intubation due to recurrent hemoptysis after pulmonary atresia operation.

The patient in case 7 who had a bilateral diaphragmatic defect combined with DORV died after abdominal wall defect repair due to complicated conditions and multi-organ failure. The patient in case 8 died at 120 days after endotracheal intubation due to serious stenosis at the lower end of the trachea near the carina, which seriously affected the ventilation and secretion discharge and led to the formation of tracheal granulation and blockage of the airway due to prolonged stimulation on the tracheal wall mucosa by the end of the endotracheal tube.

The other 10 patients were treated with cefoperazone / sulbactam combined with rifampicin, and their infections could be effectively controlled with improvement in clinical symptoms after bundle treatments such as etiological treatment and supportive treatment were also provided. Microbial clearance was achieved in cases 1,4 and 12. Ventilator support was withdrawn for these pediatric patients at 4–15 days after combined drug treatment, and they were considered cured and discharged from the hospital. The detailed data of all cases are provided in Table 1 and Supplemental Figure.

Adverse side effects included serious rash and mildly elevated aminotransferase levels during combination therapy with rifampicin in three cases. Therefore, close observation and timely withdrawal of the drug should be carried out if such effects occur.

## DISCUSSION

Acinetobacter baumannii is a non-fermentative, gram-negative bacillus that is widely distributed in nature and hospitals, and it can cause a variety of infections. According to reports in China and abroad, Ab has become the mam pathogenic bacteria for common severe infections in the ICU in the past decade (8). Because of the wide application of clinical antimicrobial agents and the development of various resistance mechanisms, Ab has changed from multi-drug sensitive to MDR and even now to deeply intractable XDR and PDR. These bacterial strains in our PCICU infection were most commonly seen for VAP followed by catheter-related bloodstream infection, followed by mediastinal infection and urinary tract infection. Due to the high degree of drug resistance of XDR/PDR Ab, the choice of clinical treatment is very difficult, which results in a high mortality rate among the infected patients. Especially in severely ill infants and young children, there are many restrictions on drug selection, resulting in extreme difficulty in choosing antimicrobial drugs and even higher mortality. Kapoor et al reported that the mortality among pediatric patients with XDR Ab infection in their PICU was 28% (3), and a hospital in eastern China reported a 30-day mortality for pediatric patients with MDR AB infection of 30% (4).

In the past 10 years, in vitro experiments and in vivo studies in animal models targeting the refractoriness of MDR-Ab were carried out worldwide. In many studies, in vitro drug sensitivity or in vivo animal experiments confirmed a very good sensitivity of XDR or PDR Ab to tigecycline, colistin, and rifampicin (9–11). However, because drug resistance to tigecycline develops easily (12) and the American FDA announced that VAP patients treated with tigecycline had a higher mortality rate in 2011 compared with that for patients treated with other drugs, its clinical efficacy remains to be observed and its application limited. Colistin has a strong antibacterial effect and the development of drug resistance is difficult. However, its long-term application is associated with a certain degree of renal toxicity and neurotoxicity (13), and colistin is relatively difficult to obtain in Mainland China. Animal experiments on pneumonia caused by XDR Ab confirmed that rifampicin can achieve the best effect in single-drug treatment, but it is susceptible to drug resistance in a short time (14–15). In view of the limitation that drug resistance occurs easily after single-drug treatment of a variety of infections caused by MDR-Ab, combination drug therapy has become a new trend due to the fact that it can reduce the generation of drug resistance and reduce the minimum inhibitory concentration (MIC) of the drug. In recent years, there have been more studies on combination therapy, and rifampicin combined with colistin, sulbactam sodium, or carbapenem, as well as carbapenem combined with sulbactam sodium have demonstrated better efficacies in animal experiments and clinical studies (2, 16).

In all pediatric patients in the present study, XDR / PDR Ab was resistant to carbapenems. Two patients had PDR Ab infection (Table 2), and the infections in the other 10 patients had certain sensitivity to combined preparations of sulbactam (sulperazone), aminoglycosides (amikacin), quinolones (levofloxacin and ciprofloxacin), or tetracycline (minocycline and tigecycline). However, quinolones and tetracyclines are not suitable for infants and young children. Sulbactam can directly and irreversibly bind the penicillin-binding proteins in the acinetobacter and thus directly kill the bacteria and maintain moderate effectiveness for some MDR Ab (17). Sulbactam is a β-lactamase inhibitor, and it alone possesses little useful antibacterial activity against most gram-positive and gram-negative organisms (useful activity against Acinetobacter baumannii). Sulbactam is only available in combination products with cefoperazone in China. Rifampicin is bactericidal and has a very broad spectrum of activity against most gram-positive and gram-negative organisms (including Acinetobacter baumannii) and specifically Mycobacterium tuberculosis. Because of rapid emergence of resistant bacteria, it must not be used alone.

The use of amikacin monotherapy for anti-MDR Ab treatment has been rarely reported, and Bernabeu-Wittel and colleagues reported that meropenem combined with amikacin did not achieve better results compared with single use of meropenem. We used sulperazone combined with amikacin for 10 days according to drug sensitivity findings in case 1, but the oxygenation index, sputum volume, lung imaging, and white blood count in this patient were not improved. When an increased dosage of sulpera-zone combined with rifampicin treatment was used, the patient’s condition was gradually improved, and the microorganisms were finally cleared from the lungs and blood (18). Another study showed that 96.7% cefoperazone/sulbactam combined with rifampin had synergistic and additive effects and reduced the MIC of rifampicin from 128 *μ*g/ml to 8 *μ*g/ml (19). Therefore, we chose the scheme of rifampicin combined with sulbactam sodium to treat Ab infection and obtained more satisfactory anti-bacterial efficacy. Notably, the adverse reactions to rifampicin include rash and liver damage, and the drugs were discontinued in case 3 when serious rashes occurred on day 4 of combined drug treatment. However, reversible recovery was achieved after the drug was discontinued in time.

The pediatric patients in our study were recovering from surgical treatment of CHD and had malnutrition, low immunological function, and pneumonia or pulmonary congestion, which was prone to causing pulmonary infection as well as heart and lung failure. In some cases, surgery was indicated because the pulmonary infection was difficult to control without treatment of the CHD. The respiratory management in patients with tracheobronchial stenosis was very difficult. For patients with syndromic CHD (with more than two extracardiac malformations), such as 21-trisomy and Noonan syndromes, the postoperative recovery time was long. Some children with complex CHD had low cardiac output syndrome after cardiac surgery, and kidney injury or even multi-organ function failure occurred in severe cases. Common features of these patients included: recovery from CHD surgery, prolonged ventilator support time, long-term retention of a central venous catheter, extended stay in the ICU, application of broad-spectrum antibiotics, malnutrition, and immune dysfunction. These are independent factors for nosocomial infection (3, 20, 21). It is very difficult to choose drugs for clinical treatment of XDR-Ab or even PDR bacteria. We used a combination therapy of rifampicin with sulbactam sodium, which effectively controlled the infection, and completely cured some patients after a sufficient treatment course.

On the basis of anti-infection treatment with drug combinations, more comprehensive bundle treatment measures are necessary to obtain good clinical outcomes for this type of critical illness in children: maintaining good heart function, ensuring organ perfusion, and creating conditions for ventilator withdrawal as soon as possible; trying to maintaining fluid balance or negative fluid balance as appropriate; adhering to scheduled feeding, maintaining intestinal function, and providing supplementary parenteral nutrition to ensure calorie supply when necessary; requiring pulmonary physical therapy to achieve good respiratory management; and administering supportive treatments such as gamma globulin to increase immunity, in order to accelerate the rehabilitation of the pediatric patients (22).

### Limitations:

The major limitations of this retrospective study were the small number of cases and the absence of controls.

## CONCLUSIONS

This accumulated experience indicates that the nosocomial infection should be prevented to the greatest extent possible. However, once VAP is caused by XDR/PDR Ab, a combined use of cefoperazone/sulbactam and rifampicin for anti-infection treatment is a good choice for infants and young children.

## Supplementary Material

1

## Figures and Tables

**Table 1.** Demographic and Clinical Characteristics.

Case	Age	Weight	Preoperative Diagnosis	High Risk Factor for Postoperative Recovery Delay	PCIS Score or Neonatal Critical Illness Score (at 24 h after surgery)	Prophase Antibiotic Treatment	Infection	Ventilator Weaning Time After Combined Treatment with Rifampicin (day)	Treatment Effect	ICU Stay (day)	Side Effect
1	7 m	5.3 kg	Severe pneumonia (with respirator), the trachea suppressed by the ectopic origin of the pulmonary artery, tracheostenosis, malnutrition	Preoperative use of antibiotics for a long time tracheostenosis	76	Ceftazidime, meropenem, cefoperazone/sulbactam combined with amikacin	VAP, CRBSI	10	Cured Microbial clearance in blood and sputum cultures	65	Rash, fade after antianaphylaxis treatment
2	2d	3.5 kg	Neonatal heavy pneumonia; TAP VC (supracardiac type)	Preoperative use of antibiotics for klebsi-ella pneumonia Delayed sternal closure	70	Meropenem	VAP	6	Cured	35	
3	40 d	3.75 kg	Severe pneumonia with respiratory function failure, VSD, severe pulmonary hypertension	Preoperative use of antibiotics for a long time Low cardiac output Acute kidney injury Peritoneal dialysis	68	Cefoperazone/sulbactam	VAP	5	Cured	28	Severe rash on the 4 days, rifampicin D/C’d
4	24 m	7 kg	Noonan syndrome, ASD	Poor postoperative immune function	92	Cefuroxime	VAP	4	Cured, Microbial clearance in sputum culture	20	
5	20 m	10 kg	ASD, VSD	Tracheotomy	96	Cefazolin	VAP	4	Cured	13	aminotransferase elevated mildly
6	12 m	6 kg	VSD, upper right tracheal bronchus, left main bronchial stenosis	Severe laryngeal edema	86	Cefuroxime, cefoperazone/sulbactam	VAP	7	Cured	19	
7	7 m	5.5 kg	Syndromic CHD Severe pneumonia DORV Bilateral diaphragm defect	Left main bronchial stenosis Left pulmonary atelectasis	68	Cefuroxime, meropenem	VAP, BSI		Died		
8	4 m	6 kg	Pulmonary artery sling, tracheostenosis, severe pneumonia (with ventilator preoperation)		90	Ceftazidime, efoperazone/sulbactam, meropenem	VAP, BSI		Died		
9	36 m	15 kg	Tricuspid regurgitation and right heart failure after Eibstein deformity correction surgery	Low cardiac output, delayed sternal closure Compression of liver on right lung	60	Ceftazidime combined with vancomycin	VAP	6	Cured	23	
10	23 m	9 kg	Tetralogy of Fallot with collateral formation and tracheal bronchus	Tracheostenosis, endotracheal granulation formation	80	Cefuroxime	VAP	4	Cured	15	
11	6 m	5 kg	Down’s syndrome	Postoperative low cardiac output	64	Cefazolin	VAP	4	Cured	12	
12	46 m	16 kg	DORV	ECMO assisted circulation for 4 days	90	Cefuroxime, cefoperazone/sulbactam	VAP	3, reintubation, 5	Improved	21	

PCIS, pediatric critical illness score; VAS, ventilator-associated pneumonia; BSI, bloodstream infection; CRBSI, catheter related bloodstream infection; ASD, atrial septal defect; VSD, ventricular septal defect; DORV, double outlet right ventricle; TAPVC, total anomalous pulmonary venous connection; PA, pulmonary atresia; D/C’d, discontinued.

PCIS (not for neonates): non-critically ill > 80, critically ill: 80–71, very critical <70. Neonatal critically ill score: non-critical ill: > 90, critical ill: 90–70, very critical ill: < 70.

**Table 2.** Results of Drug Susceptibility Testing.

Antibacterial medicines	Cephalosporin (III~IV)	Fluoroquinolones		Aminoglycosides	Tetracyclines		Co-trimoxazole
	cefoperazone / sulbactam	ciprofloxacin	levofloxacin	amikacin	minocycline	tigecycline	
Case 1	I	R	R	S	R	S	R
Case 2	R	R	R	I	I	S	R
Case 3	I	R	R	R	R	S	R
Case 4	I	R	R	R	I	S	R
Case 5	S	I	S	R	S	N	S
Case 6	R	S	S	R	R	S	I
Case 7	R	R	R	R	R	N	R
Case 8	R	R	R	R	R	N	R
Case 9	R	R	R	R	I	N	S
Case 10	I	R	S	S	S	N	S
Case 11	R	R	R	R	R	S	R
Case 12	R	S	R	R	R	S	R
		Not applicable to infants and children			Not applicable to infants and children		

Note: other drugs tested, but not listed in table 2 were: ampicillin / sulbactam, ticarcillin / clavulanic acid, aztreonam, ceftazidime, cefotaxime, cefepime, gentamicin, imipenem, meropenem, piperacillin /tazobactam.

I, intermediate; R, resistant; S, sensitivity; N, not performed.

## References

[1] Cisneros JM, Rodríguez-Bafio J. Nosocomial bacteremia due to Acinetobacter baumannii: epidemiology, clinical features and treatment. Clin Microbiol Infect 2002;8(11):687–93. 12445005 10.1046/j.1469-0691.2002.00487.x

[2] Pachón-Ibáfiez ME, Docobo-Pérez F, López-Rojas R, Domínguez-Herrera J, Jiménez-Mejias ME, García-Curiel A, Efficacy of Rifampin and Its Combinations with Imipenem, Sulbactam, and Colistin in Experimental Models of Infection Caused by Imipenem-Resistant Acinetobacter baumannii. Antimicrob Agents Chemother 2010;54(3):1165–72. 20047914 10.1128/AAC.00367-09 PMC2825983

[3] Kapoor K, Jain S, Jajoo M, Dublish S, Dabas X Manchanda V Risk Factors and Predictors of Mortality in Critically ill Children with Extensively-Drug Resistant Acinetobacter baumannii Infection in a Pediatric Intensive Care Unit. Iran J Pediatr 2014; 24(5): 569–74. 25793063 PMC4359409

[4] Fang C, Chen XJ, Zhou M. Epidemiology and Cytokine Levels among Children with Nosocomial Multidrug-Resistant Acinetobacter baumannii Complex in a Tertiary Hospital of Eastern China. PLoS One 2016;11(8):e0161690. 27579592 10.1371/journal.pone.0161690 PMC5007015

[5] Song JY, Kee SY, Hwang IS, Seo YB, Jeong HW, Kim WJ, In vitro activities of carbapenem/sulbactam combination, colistin, colistin/rifampicin combination and tigecycline against carbapenem-resistant Acinetobacter baumannii. J Antimicrob Chemother 2007;60(2):317–22. 17540672 10.1093/jac/dkm136

[6] Gauthier TP. Editorial commentary: rifampicin Plus Colistin in the Era of Extensively Drug-Resistant Acinetobacter baumannii Infections. Clin Infect Dis 2013;57(3);359–61. 23616496 10.1093/cid/cit262

[7] Saballs M, Pujol M, Tubau F, Peña C, Montero A, Dominguez MA, Rifampicin/imipenem combination in the treatment of carbapenem-resistant Acinetobacter baumannii infections. J Antimicrob Che­mother 2006;58(3):697–700. 10.1093/jac/dkl274 16895941

[8] Wong D, Nielsen TB, Bonomo RA, Pantapalangkoor P, Luna B, Spellberg B. Clinical and Pathophysiological Overview of Acinetobacter Infections: a Century of Challenge. Clin Microbiol Rev 2017;30(1);409–47. 27974412 10.1128/CMR.00058-16 PMC5217799

[9] Pachón-Ibáñez ME, Jiménez-Mejías ME, Pichardo C, Llanos AC, Pachón J. Antimicrobial activity of tigecycline (GAR-936) against multiresistant Acinetobacter baumannii. Antimicrob Agents Chemother 2004;48(11);4479–81. 15504889 10.1128/AAC.48.11.4479-4481.2004 PMC525443

[10] Falagas ME, Kasiakou SK. Colistin: the revival of polymyxins for the management of multidrug-resistant gram - negative bacterial infections. Clin Infect Dis 2005;40:1333–41. 15825037 10.1086/429323

[11] Montero A, Ariza J, Corbella X, Doménech A, Cabellos C, Ayats J, Efficacy of colistin versus beta-lactams, aminoglycosides, and rifampin as monotherapy in a mouse model of pneumonia caused by multiresistant Acinetobacter baumannii. Antimicrob Agents Chemother 2002;46:1946–52. 12019113 10.1128/AAC.46.6.1946-1952.2002 PMC127272

[12] Curcio D Treatment of recurrent urosepsis with tigecycline: a pharmacological perspective. J Clin Microbiol 2008;46:1892–3. 18460636 10.1128/JCM.02494-07 PMC2395114

[13] Molina J, Cordero E, Pachón J. New information about the Polymyxin/Colistin class of antibiotics. Expert Opin Pharmacother 2009;10:2811–28. 19929704 10.1517/14656560903334185

[14] Montero A, Ariza J, Corbella X, Doménech A, Cabellos C, Ayats J, Efficacy of colistin versus beta - lactams, aminoglycosides, and rifampin as monotherapy in a mouse model of pneumonia caused by multiresistant Acinetobacter baumannii. Antimicrob Agents Chemother 2002;46(6):1946–52. 12019113 10.1128/AAC.46.6.1946-1952.2002 PMC127272

[15] Song JY, Cheong HJ, Lee J, Sung AK, Kim WJ. Efficacy of monotherapy and combined antibiotic therapy for carbapenem-resistant Acinetobacter baumannii pneumonia in an immunosuppressed mouse model. Int J Antimicrob Agents 2009;33(1):33–9. 18835761 10.1016/j.ijantimicag.2008.07.008

[16] Motaouakkil S, Charra B, Hachimi A, Nejmi H, Benslama A, Elmdaghri N, Mohamed Benbachir Colistin and rifampicin in the treatment of nosocomial infections from multiresistant Acinetobacter baumannii. J Infect 2006;53(4):274–8. 16442632 10.1016/j.jinf.2005.11.019

[17] Levin AS. Multiresistant Acinetobacter infections: a role for sulbac- tam combinations in overcoming an emerging worldwide problem. Clin Microbiol Infect 2002;8:144–53. 12010169 10.1046/j.1469-0691.2002.00415.x

[18] Bernabeu-Wittel M, Pichardo C, García-Curiel A, Pachón-Ibáfiez ME, Ibáfiez-Martínez J, Jiménez-Mejías ME, Pharmacokinetic/pharmacodynamic assessment of the in-vivo efficacy of imipenem alone or in combination with amikacin for the treatment of experimental multiresistant Acinetobacter baumannii pneumonia. Clin Microbiol Infect 2005;11:319–25. 15760430 10.1111/j.1469-0691.2005.01095.x

[19] Xia JJ, Gong ML, Xu YP, Zhou Y, Fang XQ. Combined drug sensitivity test of carbapenem-resistant acinetobacter Baumannii (in Chinese). J Chin PLA Postgrad Med Sch 2012;33:179–81.

[20] Cisneros JM, Rodríguez-Bafio J. Nosocomial bacteremia due to Acinetobacter baumannii: epidemiology, clinical features and treatment. Clin Microbiol Infect 2002;8(11):687–93. 12445005 10.1046/j.1469-0691.2002.00487.x

[21] Leepethacharat K, Oberdorfer P. Acinetobacter baumannii Infection and colonization among pediatric patients at Chiang Mai University Hospital. J Infect Dis Antimicrob Agents 2007;24:63–73.

[22] Nimmerjahn F, Ravetch JV Anti-inflammatory actions of intravenous imunoglobulin. Annu Rev Immunol 2008;26:513–33. 18370923 10.1146/annurev.immunol.26.021607.090232

