# Supplementary material for: Combined Rifampin and Sulbactam Therapy for Multidrug-Resistant Acinetobacter Baumannii Ventilator-Associated Pneumonia in Pediatric Patients
Source: J Anesth Perioper Med. Author manuscript; Available in PMC 2019 Dec 9. (PMC6901084; doi:10.24015/JAPM.2018.0072)
Supplement: 1 [file NIHMS1007618-supplement-1.pdf]

Supplementary Appendix

This appendix has been provided by the authors to give readers additional information about their work.
Supplement to: Jinlan Chen, Yifeng Yang, Kun Xiang, David Li, Hong Liu. Combined Rifampin and Sulbactam Therapy for Multidrug-Resistant Acinetobacter Baumannii Ventilator-Associated Pneumonia in Pediatric Patients. *J Anesth Perioper Med* 2018; 5 : 176 - 185. doi: 10.24015/JAPM.2018.0072

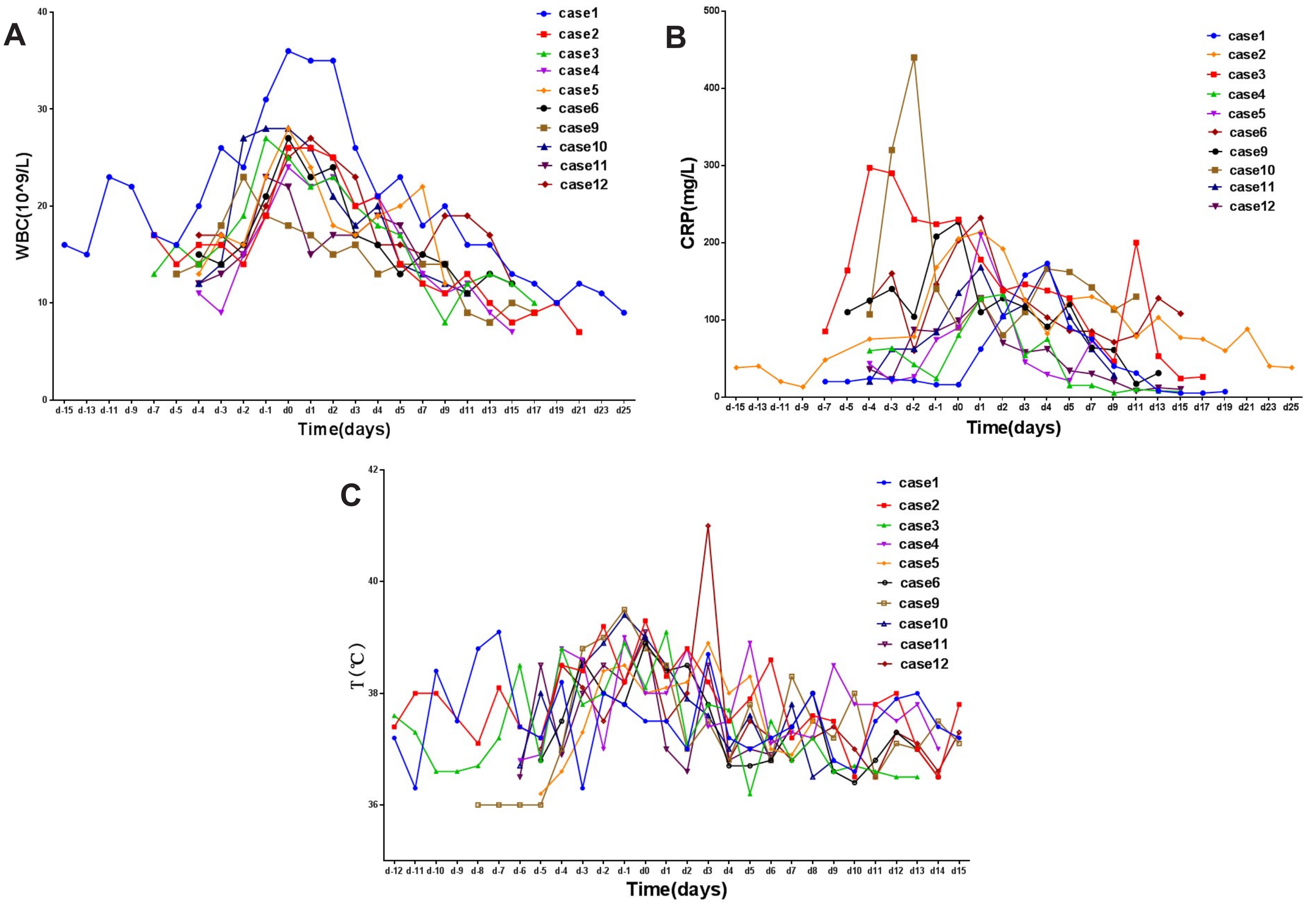

Supplemental Figure. The White Blood Cell (WBC) , C-Reactive Protein (CRP), and the temperature (T) Results of the 10 Patients Treated with Rifampicin Combined with Sulbactam Sodium through the Hospitalization (excluding 2 death cases) .
A: D0 represents the day when rifampicin combined with sulbactam sodium was started. WBC in infant with VAP caused by severe XDR-AB infection was very high before the treatment. In the first few days of this treatment, the WBC remained at high levels. But then WBC showed a downward trend, and 4 cases returned to normal eventually. **B:** D0 represents the day when treatment with rifampicin combined with sulbactam sodium was started. CRP in infant with VAP caused by severe XDR-AB infection was high (The CRP were usually high in the first 2 postoperative days because of long CPB time). CRP elevated again in case 3 after treatment stopped due to severe rash. The CRP showed a downward trend after the treatment in the rest of the cases with 3 cases returned to normal at the end. **C:** D0 represents the day when rifampicin combined with sulbactam sodium was started. Almost all cases got fever before the treatment (the temperature of case 12 was 36.0 ℃ in the first 4 days potentially due to the ECMO). The temperature of the 10 cases was gradually returned to normal after the treatment.
